# The complete mitochondrial genome of *Scythropus yasumatsui* (Coleoptera: Curculionidae)

**DOI:** 10.1080/23802359.2017.1390413

**Published:** 2017-10-17

**Authors:** Feng Zhang, Bo Hong, Zhi-Jie Chen, Yuan-Zheng Wang, Ying-Mei Li, Shu-Lian Zhang

**Affiliations:** Bio-Agriculture Institute of Shaanxi, Xi’an, China

**Keywords:** *Scythropus yasumatsui*, curculionidae, jujube, mitochondrial genomes, phylogenetic analysis

## Abstract

The complete mitochondrial genome sequence of *Scythropus yasumatsui* (Coleoptera: Curculionidae) was determined by using an Illumina platform. The circular genome was 16,472 bp in length and contained 22 transfer RNA genes (tRNAs), two ribosomal RNA genes (rRNAs), 13 protein-coding genes (PCGs), and one control region. The nucleotide composition was significantly biased (A, G, C, and T was 39.74%, 10.11%, 15.41%, and 34.74%, respectively) with A + T contents of 74.49%. All PCGs were initiated with standard ATN (ATG/ATT) codons. While 10 PCGs were terminated with TAA, two PCGs were terminated with TAG (*cytb* and *nad1*), and *nad5* was terminated with an incomplete stop codon TA. All tRNAs were predicted to contain typical cloverleaf secondary structures except *trnS1*. The phylogenetic analysis of the concatenated nucleotide sequences of 13 PCGs from 12 Curculionidae species was performed by using MrBayes 3.1.2. The results indicated that *S. yasumatsui* was more closely related to *Naupactus xanthographus* than to other species.

The bud-eating weevil, *Scythropus yasumatsui*, is native to the Northern region of China where it has become a major pest of Chinese jujube, *Ziziphus jujuba* Mill., (Huang and Li 1993). The genus *Scythropus* belongs to the family Curculionidae, but which subfamily it can be divided into is unclear. In this study, we sequenced and annotated the complete mitogenome of *S. yasumatsui* by using an Illumina platform (Coil et al. 2015). After adult specimens collected from jujube trees in Jiaxian County (38°04’N, 110°29’E), Shaanxi, China, they were deposited in the Entomological laboratory of Bio-Agriculture Institute of Shaanxi, Xi’an, Shaanxi, China.

The circular mitochondrial genome of *S. yasumatsui* was 16,472 bp in length (GenBank accession number MF807224) and contained 22 transfer RNA genes (tRNAs), 2 ribosomal RNA genes (rRNAs), 13 protein-coding genes (PCGs), and 1 control region (non-coding AT-rich region). The order and orientation of the mitochondrial genes were identical to typical mtDNA of beetles (Boore 1999). Besides the AT-rich region (1377 bp), there were 16 intergenic spacer regions ranging in size from one to 382 (516 bp in total) and 14 overlapping regions (93 bp in total) throughout the whole genome.

The nucleotide composition was significantly biased (A, G, C and T was 39.74%, 10.11%, 15.41%, and 34.74%, respectively) with A + T contents of 74.49%. All PCGs were initiated with typical ATN codons (eight with ATT and five with ATG). Whereas 10 PCGs were terminated with TAA, two PCGs were terminated with TAG (*cytb* and *nad1*), and *nad5* was terminated with an incomplete stop codon TA. All of the 22 tRNAs were predicted to contain typical cloverleaf secondary structures except the gene *trnS1* (AGN), whose DHU arm was replaced by a simple loop. *rrnL* was 1315 bp in length with A + T contents of 78.86%, and *rrnS* was 785 bp in length with A + T contents of 78.22%.

To further understand the mitogenome of *S. yasumatsui* and study the evolution of genus *Scythropus*, the concatenated nucleotide sequences of 13 PCGs from 12 Curculionidae species and outgroup species from the family Tenebrionidae (*Tribolium castaneum*) were used for the phylogenetic analysis. For the 12 Curculionidae species, they came from eight subfamilies or tribes, including Dryophthorinae (*Sitophilus oryzae*, *Sitophilus zeamais*, *Rhynchophorus ferrugineus,* and *Sphenophorus* sp.), Cryptorhynchinae (*Eucryptorrhynchus brandti* and *Eucryptorrhynchus chinensis*), Entiminae (*Naupactus xanthographus*), Curculioninae (*Curculio davidi*), Cyclominae (*Aegorhinus superciliosus*), Molytinae (*Hylobitelus xiaoi*), Platypodinae (Platypodinae sp.), and Scolytinae (Scolytinae sp.), and their complete mitochondrial sequences were obtained from GenBank of NCBI. Bayesian analyses were performed with MrBayes 3.1.2 (Ronquist and Huelsenbeck 2003), and the BI phylogenetic tree showed that *S. yasumatsui* was more closely related to *Naupactus xanthographus* than to other species (Figure 1), which meant that there was a closest relationship between *S. yasumatsui* and species from the subfamily Entiminae.

**Figure 1.**
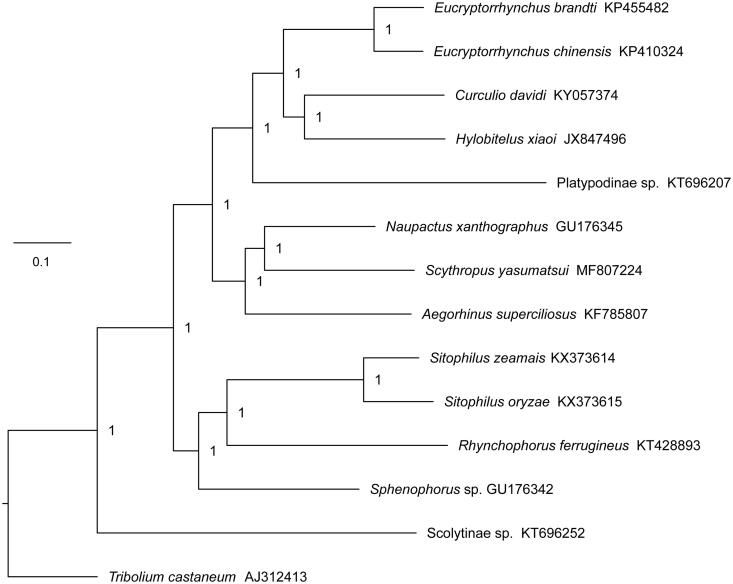
The BI phylogenetic tree of *S. yasumatsui* based on the concatenated nucleotide sequences of 13 PCGs. Above the branches were Bayesian posterior probabilities values.
